# Understanding Well-Being in Daily Life: Informal Social Support, Depression Risk, and Life Satisfaction Among Chinese Older Adults with Disabilities

**DOI:** 10.3390/bs16071216

**Published:** 2026-07-17

**Authors:** Xinzhe Wang, Chenrui Zhang, Wenhu Xu, Shiyu Chen, Gong Chen

**Affiliations:** 1 Institute of Population Research, Peking University, No.5 Yiheyuan Road, Haidian District, Beijing 100084, China; 2501213174@stu.pku.edu.cn (X.W.); zhangchenrui@pku.org.cn (C.Z.); 2401111475@stu.pku.edu.cn (W.X.); 2 College of Social Development, Hunan Women’s University, No.160 Zhongyi First Road, Changsha 410004, China

**Keywords:** life satisfaction, informal social support, depression risk, older adults with disabilities, KHB decomposition

## Abstract

Against the backdrop of population aging, the well-being of older adults with disabilities in daily life and its underlying mechanisms has increasingly become an important research topic. Using data from the 2020 wave of the China Health and Retirement Longitudinal Study (CHARLS), this study focuses on 509 individuals aged 60 and above with disabilities. It incorporates informal social support, depression risk, and life satisfaction into a unified analytical framework, and employs ordered Logit models, binary Logit models, and the KHB decomposition method to examine their interrelationships and underlying mechanisms. The results show that: (1) informal social support is positively associated with the life satisfaction of older adults with disabilities; (2) informal social support is negatively associated with depression risk; and (3) depression risk is negatively associated with life satisfaction and plays a significant mediating role in the relationship between informal social support and life satisfaction, accounting for 23.25% of the total effect. From a daily life perspective, this study reveals that the well-being of older adults with disabilities is not solely derived from institutional resource provision, but is generated and sustained through ongoing social interactions and relational networks. The study therefore deepens our understanding of the everyday and relational dimensions of subjective well-being in later life.

## 1. Introduction

At present, China has the largest older population in the world and is among the countries experiencing a relatively rapid process of population aging. By the end of 2024, the population aged 60 and above in China had reached 310 million, accounting for 22.0% of the total population; the population aged 65 and above had reached 220 million, accounting for 15.6% of the total population. It is projected that from 2025 to 2050, the disability rate among China’s population aged 65 and above will show an overall phased upward trend, ranging from 12.27% to 15.05%, while the number of older adults with disabilities will continue to increase from approximately 25.90 million to 58.64 million (Zhang et al., 2025). The expansion of the population of older adults with disabilities poses major challenges to China’s social and economic development, particularly in terms of how to ensure their quality of later life and improve their well-being in daily life.

As an important psychological indicator involving both affective and cognitive dimensions, subjective well-being includes individuals’ cognitive evaluations of their life circumstances as well as their affective experiences (Diener, 1984). Among these components, life satisfaction represents the cognitive evaluative dimension of subjective well-being. It reflects individuals’ overall judgment of their quality of life based on their own life experiences, value standards, and actual living conditions, and has shown good convergent validity with other forms of subjective well-being assessment (Pavot & Diener, 2009). Affective experience is also an important component of subjective well-being and mainly involves positive and negative emotions (Fors Connolly & Gärling, 2024). Although life satisfaction and affective experience correspond respectively to the cognitive evaluative and affective dimensions of subjective well-being, they are not independent of each other. According to feelings-as-information theory, individuals often use their persistent emotional states as an important source of information when evaluating their life circumstances. Therefore, both positive and negative emotional experiences may participate in the formation of life satisfaction judgments (Schwarz, 2012). Existing studies have more often understood this relationship from the perspective of the positive role of positive emotions in promoting life satisfaction. Research has shown that both positive and negative emotions influence individuals’ life satisfaction judgments, but the association between positive emotions and life satisfaction is usually stronger than that between negative emotions and life satisfaction (Busseri, 2018; Kuppens et al., 2008), while the independent effect of negative emotions is relatively weak or unstable (Jovanović & Joshanloo, 2022). This does not mean that negative emotions are unimportant; rather, it suggests that the psychological consequences of negative emotions should be understood in relation to specific populations and living contexts.

For older adults with disabilities, the meaning of negative emotions needs to be understood particularly in the context of daily life stress and functional limitations. Temporary sadness, anxiety, or worry does not necessarily indicate impaired mental health. However, when negative emotions and experiences such as loneliness, helplessness, anxiety, and sadness persist over time, and when positive emotions such as hopefulness and happiness are also noticeably lacking, these emotional experiences may accumulate into a higher risk of depression. Older adults with disabilities are more likely to face continuous stress and relational isolation due to functional limitations, reduced autonomy in daily life, increased dependence on care, and fewer opportunities for social participation. Therefore, this study focuses on depression risk as a comprehensive psychological manifestation of sustained negative emotional burden and insufficient positive emotion, and treats it as an important mental health dimension linking daily support resources and life satisfaction among older adults with disabilities.

In this sense, informal social support may constitute an important everyday resource for understanding life satisfaction among older adults with disabilities. As a vulnerable group with limited ability to perform activities of daily living, older adults with disabilities often rely more heavily on informal social support provided by family members, relatives, friends, neighbors, and community relationships. Such support includes not only instrumental support in basic daily activities such as dressing, bathing, and eating, but also emotional support that helps maintain their connection with the outside world through companionship, communication, emotional responsiveness, and social interaction. According to the stress-buffering theory, social support can reduce the adverse effects of stressful events on individuals’ psychological states and well-being by providing practical assistance, emotional comfort, and coping resources (Cohen & Wills, 1985). For older adults with disabilities, informal support from family members, friends, neighbors, and the community can alleviate the practical pressures arising from limitations in daily activities through care services, while also reducing feelings of loneliness and the risk of depression by providing companionship, communication, emotional support, and opportunities for social participation.

In the Chinese context, studying informal social support among older adults with disabilities has particular theoretical and practical significance. Older adult care in China has long been family-based, with spouses, children, and other relatives playing core roles in daily care, economic support, and emotional comfort. Influenced by filial piety and intergenerational responsibility, family care for older adults with disabilities is not merely a living arrangement, but also carries strong moral obligations and cultural expectations. However, as the long-term effects of the one-child policy become increasingly visible, family size continues to shrink, population mobility intensifies, and women’s labor force participation rises, traditional family care resources are under growing pressure. On the one hand, older adults with disabilities have increasing needs for daily care and emotional companionship; on the other hand, the number of family members and the amount of time available to share caregiving responsibilities are relatively declining. At the same time, China’s formal long-term care services and community-based care system are still developing and improving. Therefore, studying the relationships among informal social support, depression risk, and life satisfaction not only helps deepen theoretical understanding of the formation mechanism of subjective well-being among older adults with disabilities, but also provides empirical evidence for family care, community support, and the construction of long-term care service systems.

### 1.1. Understanding Life Satisfaction Among Older Adults with Disabilities from the Perspective of Daily Life

Life satisfaction refers to a comprehensive subjective evaluation that individuals make of their current living conditions based on their own value standards and life experiences. It is not a one-dimensional or momentary feeling, but rather a psychological assessment system that integrates multiple factors, including material conditions, interpersonal relationships, and self-realization. It is reflected in the extent to which individuals maintain a sustained sense of approval toward their quality of life. Older adults’ evaluations of life are often gradually formed and adjusted through social interaction, activity participation, relationship maintenance, and emotional experience. Related studies have also shown that daily activities and social interactions constitute important foundations closely associated with subjective well-being and life satisfaction (Keyes et al., 2024; Wild et al., 2025).

This is particularly salient among older adults with disabilities. Functional limitations not only indicate a decline in the ability to perform basic activities of daily living, but also imply a reduced range of activity, fewer opportunities for social contact, and greater dependence on external assistance. Existing research has demonstrated a stable association between disability, lower quality of life, and poorer subjective well-being (Wilk et al., 2024). Among older adults in China, activities of daily living, depressive symptoms, and life satisfaction have also been found to be significantly related (Liao et al., 2024). A study of Chinese older adults further found that community support, social isolation, and loneliness jointly shape their life satisfaction (Jiang et al., 2024).This implies that life satisfaction reflects an individual’s multidimensional assessment of themselves, encompassing but not limited to evaluations of objective health status and material conditions, living circumstances, as well as the support and social connections available in daily life. Therefore, using life satisfaction as a proxy variable for well-being is justified for further understanding the sense of well-being in the daily lives of older adults with disabilities.

### 1.2. Informal Social Support: A Key Everyday Resource for Older Adults with Disabilities

Informal social support refers to the resources individuals receive in daily life through non-institutionalized relational networks, primarily derived from familiar relationships such as family members, friends, relatives, neighbors, and community members. Compared to formal support provided by government agencies or professional organizations, informal social support is characterized by its everyday nature, relationality, and non-contractual nature. Unlike institutional assistance offered by governmental bodies or specialized organizations, the informal support system emphasizes reciprocity in interpersonal interactions and serves as a buffer against crises, stress, and negative emotions through informal communication channels. Considering the living circumstances of older adults with disabilities, this study examines informal social support from two perspectives: first, instrumental support, which encompasses practical care and assistance received in daily life; second, emotional support, derived from social participation and interpersonal interactions as sources of emotional comfort and relational support.

Existing studies indicate that informal social support for older adults with disabilities is a social support resource centered on the family and extended to relatives, friends, and neighbors. In China, the vast majority of older adults with disabilities still live at home, and families continue to assume the most fundamental and sustained responsibilities in care-giving (Peng, 2024). Although care-giving structures vary across countries, spouses and children remain the primary sources of support for older adults with disabilities. Men tend to rely more on their spouses, whereas women are more often cared for primarily by their children (Jain & Sheehan, 2023). Among rural older adults with disabilities in China, informal family care continues to occupy a central position, while relatives, neighbors, and communities play more supplementary roles (Gao, 2015; L. Wang, 2013). This suggests that the support networks of older adults with disabilities are not merely substitutes for institutional provision, but constitute an important foundation for maintaining their daily lives.

The availability of informal social support also varies according to individual circumstances and relational conditions. Research has shown that spatial proximity between family members and older adults, the quality of relationships, and caregivers’ capacity to provide support all influence the level of informal support available (Akosile et al., 2024; Bražinová & Chytil, 2024). Within Chinese communities, older adults with disabilities who are younger and have more children tend to receive higher levels of support, whereas a greater number of chronic conditions and more severe disability can reduce the social support available to them (Pan et al., 2024). This suggests that informal social support is dynamically shaped by family structure, health status, and relational networks.

The role of informal social support extends beyond instrumental assistance such as care provision, also manifesting in relationship maintenance and emotional interaction. Evidence from older adults disabled by fractures indicates that informal support helps restore social participation and maintain a more active lifestyle (Ekström et al., 2013). Studies in China further show that social support can significantly reduce loneliness among older adults, with family support playing the most important role (T. Dong, 2017). When communication within families is more frequent, adult children show greater respect for older adults’ opinions, and community services are more adequately provided, loneliness among older adults with disabilities declines significantly (Liu et al., 2018). Improvements in social support and reductions in depressive symptoms have also been identified as important conditions for enhancing mental health and quality of life among older adults with disabilities (Gu et al., 2017). Therefore, informal social support serves not only as an instrumental resource for older adults with disabilities to maintain daily life but also as a crucial emotional resource for sustaining relational connections, obtaining emotional responses, and forming positive life evaluations, thereby influencing their life satisfaction and well-being. Based on this, this study proposes:

**Hypothesis** **H1.** *Informal social support in daily life is positively associated with the life satisfaction of older adults with disabilities in China.*

### 1.3. Depression Risk: A Psychological Pathway Linking Informal Social Support and Life Satisfaction

Depression risk is a significant manifestation of mental health risks among older adults with disabilities, typically characterized by persistent negative emotional experiences, insufficient positive emotions, sleep disturbances, feelings of loneliness and helplessness, as well as reduced sense of control over life—symptoms all associated with depression. It should be noted that the depression risk discussed in this article is not equivalent to a clinical diagnosis of depression but rather refers to a psychological risk state where individuals exhibit elevated levels of depressive symptoms on assessment scales. For older adults with disabilities, declining physical function implies limitations in daily activities and may lead to diminished autonomy and increased dependence on care, thereby further increasing their susceptibility to depression.

Research suggests that living arrangements, household relationships, and support environments are closely related to depression risk, whereas informal social support can significantly improve the mental health of older adults (Y. Dong et al., 2024; Jia et al., 2023). Although studies using depression risk as a mediating variable have begun to emerge in gerontological research, they remain limited and focus primarily on the relationship between social support and life satisfaction, with far less attention paid to older adults with disabilities and their informal social support. Depression risk is itself an important determinant of life satisfaction. Depression has been shown to significantly reduce subjective well-being, and clear mediating and interactive relationships have been reported among ADL, depressive symptoms, and life satisfaction (Liao et al., 2024; W. Wang et al., 2026). This indicates that the risk of depression not only reflects the mental health risks faced by older adults with disabilities but may also constitute a significant psychological pathway explaining the formation process of their life satisfaction.

Based on this, this study proposes:

**Hypothesis** **H2.** *Informal social support in daily life is negatively correlated with the depression risk of older adults with disabilities in China.*

**Hypothesis** **H3.** *The risk of depression is negatively correlated with the life satisfaction of older adults with disabilities in China and plays a mediating role between informal social support and life satisfaction.*

**Figure 1:**
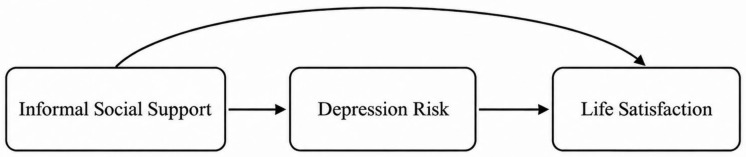
Conceptual Framework of the Relationship among Informal Social Support, Depression Risk, and Life Satisfaction.

Although existing research has separately examined the relationship between social support, depression risk, and life satisfaction among the elderly, three issues remain worthy of further investigation: First, current studies predominantly explain elderly well-being from the perspectives of health, economic, or institutional support, with limited direct exploration of how “informal social support” continuously shapes well-being. Second, there is scarce research and discussion on the internal relationship between the cognitive evaluative and affective dimensions of well-being. Finally, while the role of depression risk between social support and life satisfaction has been partially addressed, empirical evidence surrounding the chain of “informal social support—depression risk—life satisfaction” among older adults with disabilities remains relatively limited. Based on this, this study focuses on older adults with disabilities in China to examine the association between informal social support in daily life and life satisfaction, and further analyzes the mediating role of depression risk, aiming to provide empirical evidence from the Chinese context for understanding well-being in daily life during old age (Figure 1).

## 2. Materials and Methods

### 2.1. Data Source and Sample

This study uses data from the 2020 wave of the China Health and Retirement Longitudinal Study (CHARLS), a large interdisciplinary survey initiated by Peking University and implemented in collaboration with Wuhan University. CHARLS was designed to collect nationally representative micro-level data on Chinese middle-aged and older adults and their households and has been widely used in research on aging, health, and socioeconomic conditions. The “Health Status and Functioning” module contains information on respondents’ health status, physical functional limitations, caregivers, cognition, and depressive symptoms, thereby providing appropriate data for measuring disability, depression risk, and support-related variables.

### 2.2. Measures

#### 2.2.1. Informal Social Support

This study does not conceptualize informal social support as a single type of care resource, but rather as comprehensive support available to older adults with disabilities when integrated into their daily relational networks, encompassing both the number of caregivers and social participation scores. The number of caregivers reflects the accessibility of instrumental support, primarily pertaining to daily care and life assistance. We employed auxiliary variables derived from the CHARLS Questionnaire—specifically those assessing “whether assistance was received in dressing, bathing, eating, getting up, using the toilet, household chores, cooking, shopping, making phone calls, taking medication, or managing finances”—to statistically analyze and recode existing supporter data. Specifically: no assistance = 0; 1–2 assistants = 1; 3–4 assistants = 2; 5–6 assistants = 3; ≥7 assistants = 4. Social participation measures relational integration and interaction opportunities, representing emotional support within informal social networks. This was achieved by combining questions such as “Have you engaged in the following social activities during the past month?” and “How frequently do you perform these activities? Approximately daily, weekly, or infrequently?” to derive activity participation frequency scores categorized into five levels: 0, 1–2, 3–4, 5–6, 7–8 points. These scores were then aggregated per item to form an overall social participation score, which serves as an indicator of the social network and emotional support received by older adults with disabilities within community-based activities.

The combination of these two measures collectively reflects the informal social support available to older adults with disabilities in daily life. To mitigate the impact of outliers and skewness on the results, a logarithmic transformation was applied to informal social support data to reduce skewness and accommodate zero values, thereby meeting the distribution requirements for the regression model.

#### 2.2.2. Life Satisfaction

The dependent variable in this study is the life satisfaction of older adults with disabilities, measured using the question “Overall, are you satisfied with your life?” from the CHARLS 2020 questionnaire. For clarity, the original response options were reversed-coded to a scale ranging from 1 to 5, where “1” indicates “not at all satisfied”, “2” indicates “not very satisfied”, “3” indicates “somewhat satisfied,” “4” indicates “very satisfied,” and “5” indicates “completely satisfied.” Higher scores correspond to greater life satisfaction.

#### 2.2.3. Depression Risk

CHARLS 2020 employs the Brief Version of the Depression Scale from the Epidemiology Research Center (CES-D10) to assess respondents’ emotional experiences over the past week. The scale comprises 10 items covering aspects such as distress, difficulty concentrating, low mood, fatigue in performing tasks, optimism about the future, fear, poor sleep quality, happiness, loneliness, and inability to continue living normally, providing a comprehensive measure of both positive and negative emotional impacts in older adults. Each item is scored based on symptom frequency: “rarely or never” receives a score of 0, “not often” scores 1, “sometimes or about half the time” scores 2, and “most of the time” scores 3. Items related to positive emotions—such as “optimism about the future” and “feeling happy”—are scored inversely.

The CES-D10 total score ranges from 0 to 30, with higher scores indicating greater risk of depression. Based on existing research, this study defines a total score ≥ 10 as indicative of depression risk and constructs corresponding variables accordingly. The coding scheme is as follows: 0 = no depression risk; 1 = have depression risk.

#### 2.2.4. Control Variables

The control variables included age, gender, hukou status (agricultural vs. non-agricultural), educational attainment (junior high school or below, technical secondary school/high school, and college or above), place of residence (urban vs. rural), and marital status (married but living apart, married and cohabiting, and unmarried/living apart/divorced/widowed).

### 2.3. Statistical Analysis

This study employed Stata 18.0 to process and analyze the CHARLS 2020 data. Prior to formal analysis, the data underwent cleaning and preprocessing: samples aged under 60, individuals without disability, as well as those with missing or invalid responses regarding life satisfaction, the 10-item Center for Epidemiologic Studies Depression Scale (CES-D10) depression scale scores, informal social support, and control variables were excluded, resulting in a sample of 509 older adults aged 60 and above with disabilities. Statistically, descriptive statistics were first used to characterize demographic characteristics and core variables, including baseline demographics, disability status, levels of informal social support, distribution of life satisfaction, and depression risk profiles. Correlation analyses were conducted to examine relationships among core variables. Given that life satisfaction is a five-category ordered variable and depression risk is a binary variable, an ordered Logit model was employed to analyze the relationship between informal social support and life satisfaction among older adults with disabilities, while a binary Logit model was used to assess the impact of informal social support on depression risk. The depression risk variable was subsequently incorporated into the analytical framework to investigate its mediating role between informal social support and life satisfaction. For mediation effect testing, the KHB decomposition method was applied to distinguish between total effects, direct effects, and indirect effects, thereby avoiding model bias across different analytical models. Through the above analysis, this study aims to elucidate how informal social support in daily life influences the life satisfaction of older adults with disabilities, as well as the role of depression risk in this process.

## 3. Results

### 3.1. Descriptive Characteristics of the Sample

Among the 509 older adults with disabilities, the sample had an average age of 70.56 years; females accounted for 57.76%, those with agricultural household registration made up 74.46%, rural residents represented 68.57%, and married cohabitants constituted 70.92%. Regarding life satisfaction, the most common responses were “Somewhat Satisfied” (44.20%) and “Very Satisfied” (28.29%); no depression risk was reported by 29.47% of respondents. Overall, the sample primarily consisted of older adults with disabilities who were advanced in age, residing in rural areas, and living in marital relationships, with their life satisfaction levels generally moderate (Table 1).

### 3.2. Correlation Analysis

Table 2 presents the correlation analysis results among the core variables. Informal social support shows a significant positive correlation with life satisfaction (r = 0.1173, *p* < 0.05), indicating that higher levels of informal social support are associated with greater life satisfaction among older adults with disabilities. Depression risk exhibits a significant negative correlation with life satisfaction (r = −0.2792, *p* < 0.05), suggesting that older adults with disabilities at risk of depression have relatively lower life satisfaction. In contrast, informal social support is negatively correlated with depression risk (r = −0.0782), but this association is not statistically significant, indicating that its relationship remains weak without controlling for other variables and warrants further investigation.

### 3.3. Regression Analysis

To further clarify the relationship between informal social support and life satisfaction among older adults with disabilities, this study incorporates demographic variables—including age, gender, household registration type, education level, place of residence, and marital status—as control variables, and employs an ordered Logit model to examine the association between informal social support and life satisfaction.

The results are presented in Table 3. The regression coefficient for age was β = 0.046, *p* = 0.001, indicating a positive correlation between age and life satisfaction among older adults with disabilities. This may be attributed to psychological adaptation to their living conditions, adjustment of expectations, or accumulation of existing social resources; however, this explanation requires further validation in subsequent studies. Upon incorporating informal social support into Model 1, the R^2^ value in Model 2 increased from 0.014 to 0.019. The regression coefficient for informal social support was positive and significant (β = 0.477, *p* = 0.018), suggesting that higher levels of informal social support correlate with greater life satisfaction among older adults with disabilities, thereby supporting Hypothesis H1.

Table 4 further presents the binary Logit regression results regarding the impact of informal social support on depression risk. Age exhibits a negative correlation with depression risk among older adults with disabilities (β = −0.040, *p* < 0.05), whereas gender shows a positive correlation (β = 0.560, *p* < 0.01). Upon incorporating the informal social support variable, the regression coefficient for age slightly increased (β = −0.039, *p* < 0.05), and the coefficient for female gender rose to β = 0.621 in Model 2 (*p* < 0.01), indicating that the depression risk is further elevated among female older adults with disabilities compared to males. The model’s explanatory power improved from R^2^ = 0.036 in Model 1 to R^2^ = 0.043 in Model 2, with the regression coefficient for informal social support reaching β = −0.499 (*p* < 0.05), supporting Hypothesis H2. Notably, after controlling for informal social support, the coefficient for female older adults with disabilities increased from β = 0.560 to β = 0.621 while remaining statistically significant, suggesting that their depression risk did not diminish but rather intensified despite the inclusion of this variable. This implies that depression risk among female older adults with disabilities may be influenced not only by the quantity of support resources but also by factors such as support quality, marital status, and role-related stress.

### 3.4. Mediation Analysis

Table 5 presents the results of mediation path tests after incorporating depression risk into the model. Model 1, controlling for demographic variables, examined solely the association between depression risk and life satisfaction. The results showed a significant negative correlation between depression risk and life satisfaction (β = −1.211, *p* < 0.001), indicating that older adults with depression risk experience lower life satisfaction. In Model 2, which added informal social support to Model 1, the strength of the association between depression risk and life satisfaction slightly weakened, yet the regression coefficient remained significantly negative (β = −1.183, *p* < 0.001). Meanwhile, the regression coefficient for informal social support was positive but only marginally significant (β = 0.385, *p* = 0.059). These findings suggest that when depression risk is included in the analytical framework, the correlation between informal social support and life satisfaction diminishes, implying that depression risk may play a significant mediating role between the two variables.

**Figure 2:**
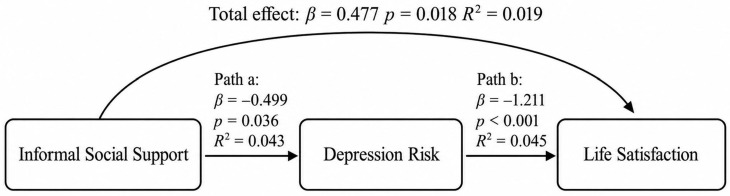
Mediation Effect of Depression risk between Informal Social Support and Life Satisfaction among Older Adults with disabilities.

On this foundation, Table 6 further decomposes the mediating effects using the KHB method. The results indicate that the total effect of informal social support on life satisfaction is 0.5010, suggesting that higher levels of informal social support correlate with greater life satisfaction among older adults with disabilities. After accounting for depression risk, the direct effect of informal social support on life satisfaction is 0.3845, though only marginally significant. The indirect effect measures 0.1165 and reaches statistical significance (*p* = 0.042), with a 95% confidence interval of [0.0043, 0.2286] excluding zero, confirming the mediative role of depression risk. This indirect effect accounts for 23.25% of the total effect, suggesting that approximately 23.25% of the relationship between informal social support and life satisfaction can be explained through the psychological pathway of depression risk. Thus, depression risk plays a partial mediating role between informal social support and life satisfaction, supporting Hypothesis H3 (Figure 2).

## 4. Discussion

This study examines the relationship between informal social support, depression risk, and life satisfaction among older adults with disabilities in China from a daily life perspective. Overall, the findings support the hypotheses proposed in this study and further demonstrate that informal support in daily life is not only a crucial resource for maintaining basic care but also closely associated with their mental health status and life evaluation: First, informal social support is positively correlated with life satisfaction among older adults with disabilities, supporting Hypothesis H1. This indicates that, after controlling for age, gender, household registration type, education level, place of residence, and marital status, older adults with disabilities who receive greater informal social support tend to exhibit higher life satisfaction. This finding aligns well with existing research on social support and elderly well-being, confirming that social relationships and support resources are significant factors influencing subjective life evaluations among older adults. Second, the study also revealed a negative correlation between informal social support and depression risk among older adults with disabilities, with depression risk serving as a partial mediator between informal social support and life satisfaction, thereby supporting Hypotheses H2 and H3. KHB decomposition results further show that depression risk accounts for approximately 23.25% of the association between informal social support and life satisfaction. Informal social support may influence life satisfaction not only through links with daily support resources but also through its psychological pathway of reducing depression risk.

This study contributes to a deeper understanding of the well-being of older adults with disabilities in daily life: First, starting from everyday contexts, this study integrates informal social support, depression risk, and life satisfaction into a unified analytical framework, thereby expanding our comprehension of the mechanisms underlying subjective well-being in older adults. Second, regarding the internal structure of subjective well-being, cognitive evaluation dimensions and emotional experience dimensions are often examined separately rather than within a shared explanatory framework. This study employs life satisfaction as a proxy indicator for the cognitive evaluation dimension of subjective well-being among older adults with disabilities, while using depression risk as a comprehensive representation of persistent negative emotional burdens and insufficient positive emotions at the mental health level, aiming to establish a connection between “life appraisal” and “emotional experience.” This approach facilitates the shift in well-being from a macro-level metric to a micro-level aspect of daily living, enabling a more accurate assessment of the actual living conditions of older adults with disabilities. Consequently, the well-being of older adults with disabilities should not be narrowly understood as a function of social resource availability or health status but rather as a subjective experience that is continuously generated, maintained, and adjusted throughout daily life. In the Chinese context, the well-being of older adults with disabilities is often intertwined with traditional family-based care practices and filial culture. Filial culture not only shapes children’s moral responsibility to care for their parents but also influences older adults’ subjective perceptions of being cared for and accepted by their families. Thus, for older adults with disabilities in China, informal social support serves not merely as a supplement to external care resources but also as a culturally significant form of relational affirmation.

Based on these findings, enhancing the well-being of older adults with disabilities cannot rely solely on formal care services; it also requires strengthening psychological support, informal social networks, and opportunities for social engagement at the daily living level. First, depression risk screening and psychological support should be integrated into community-based elderly care services and family care support systems. Through regular visits, psychological counseling, and caregiver support, negative emotions such as loneliness, helplessness, and depression among older adults with disabilities can be promptly identified and alleviated. Second, informal support networks comprising families, neighbors, volunteers, and community organizations should be further strengthened to reduce caregiving pressures while providing stable companionship, communication, and emotional support. Finally, more inclusive social participation environments should be created at the community level—through improved accessibility facilities, organized interest-based activities tailored to their physical conditions, and promotion of neighborhood mutual aid and intergenerational interaction—allowing older adults with disabilities not only to remain cared-for but also to regain relational connections, a sense of purpose, and control over their lives through meaningful participation.

This study has several limitations: First, it uses cross-sectional data, which allows us to identify statistical associations and mediating pathways but does not support rigorous causal inference; Second, informal social support was measured by combining caregiver availability and social participation. Although this approach covers both instrumental and emotional support in daily life, these two dimensions may represent partially heterogeneous forms of support and may have different associations with well-being; Third, the explanatory power of the regression models was relatively limited. This suggests that life satisfaction and depression risk among older adults with disabilities may also be influenced by other factors not included in this study; Fourth, after controlling for informal social support, the results show that depression risk was more pronounced among female older adults with disabilities. However, this study did not further examine the interaction between gender and informal social support.

Future research can be further expanded in three directions: First, longitudinal data should be used to examine the dynamic relationships among informal social support, depression risk, and life satisfaction, thereby strengthening causal inference; Second, future studies could distinguish between different sources and types of informal support, particularly instrumental and emotional support, to clarify their potentially different roles in shaping well-being; Third, more attention should be paid to female older adults with disabilities by examining how gender roles, marital status, caregiving experiences, and family responsibilities jointly influence their depression risk and life satisfaction.

## 5. Conclusions

Based on CHARLS 2020 data, this study analyzes the relationship between informal social support, depression risk, and life satisfaction among older adults with disabilities in China. The findings indicate that higher levels of informal social support are associated with greater life satisfaction and lower depression risk among these individuals; conversely, higher depression risk correlates with lower life satisfaction. KHB mediation analysis results demonstrate that depression risk plays a significant mediating role between informal social support and life satisfaction, accounting for approximately 23.25% of the total effect. These results suggest that informal social support contributes to the formation of well-being in older adults with disabilities by reducing depression risk and enhancing life evaluation. Future efforts should focus on improving family care support systems, community engagement platforms, and mental health screening mechanisms to further enhance the daily well-being of this population.

## Figures and Tables

**Table 1:** Descriptive Data of Study Variables.

Category	Subcategory	N	Percentage
Gender	Male	215	42.24%
	Female	294	57.76%
Household Registration Type	Agricultural	379	74.46%
	Non-Agricultural	130	25.54%
Place of Residence	Rural	349	68.57%
	Urban	160	31.43%
Marital Status	Unmarried, Living Apart, Divorced, Widowed	139	27.31%
	Married but Living Apart	9	1.77%
	Married and Cohabiting	361	70.92%
Education Level	Junior High School or Below	468	91.94%
	Technical Secondary School or High School	31	6.09%
	College Degree or Above	10	1.96%
Instrumental Support	0 = No caregiver	51	10.02%
	1 = 1–2 caregivers	312	61.30%
	2 = 3–4 caregivers	72	14.15%
	3 = 5–6 caregivers	36	7.07%
	4 = 7 or more caregivers	38	7.47%
Emotional Support	0 = No participation	332	65.23%
	1 = Score of 1–2	97	19.06%
	2 = Score of 3–4	66	12.97%
	3 = Score of 5–6	12	2.36%
	4 = Score of 7–8	2	0.39%
Informal Social Support	log[(Instrumental Support Score+ Emotional Support Score) + 1]	509	100%
Life Satisfaction	Not at All Satisfied	41	8.06%
	Not Very Satisfied	75	14.73%
	Somewhat Satisfied	225	44.20%
	Very Satisfied	144	28.29%
	Completely Satisfied	24	4.72%
Depression Risk	No Depression Risk	150	29.47%
	Have Depression Risk	359	70.53%

**Table 2:** Correlations among Informal Social Support, Life Satisfaction, and Depression Risk.

Variables	Informal Social Support	Life Satisfaction	Depression Risk
Informal Social Support	1		
Life Satisfaction	0.1173 *	1	
Depression Risk	−0.0782	−0.2792 *	1

Note: N = 509, * *p* < 0.05.

**Table 3:** Ordered Logit Regression Results for Life Satisfaction.

Explanatory Variables	Model 1		Model 2	
	β	*p*	β	*p*
Gender (Male = 0)				
Female	0.275	0.114	0.222	0.207
Household Registration Status (Agricultural = 0)				
Non-agricultural	0.100	0.635	0.118	0.573
Educational Attainment	−0.089	0.720	−0.073	0.771
Place of Residence (Rural = 0)				
Urban	−0.219	0.293	−0.221	0.291
Age	0.046 ***	0.001	0.045 ***	0.001
Marital Status (Unmarried, Living Apart, Divorced, Widowed = 0)				
Married but Living Apart	0.516	0.430	0.583	0.417
Married and Cohabiting	−0.043	0.837	−0.009	0.996
Informal Social Support			0.477 *	0.018
N	509		509	
R^2^	0.014		0.019	

Note: Model 1 includes only control variables, whereas Model 2 additionally includes informal social support. * *p* < 0.05, *** *p* < 0.001.

**Table 4:** Logit Regression Results for Depression Risk.

Explanatory Variables	Model 1		Model 2	
	β	*p*	β	*p*
Gender (Male = 0)				
Female	0.560 **	0.007	0.621 **	0.003
Household Registration Status (Agricultural = 0)				
Non-agricultural	−0.273	0.319	−0.280	0.31
Educational Attainment	−0.293	0.296	−0.316	0.261
Place of Residence (Rural = 0)				
Urban	−0.048	0.852	−0.058	0.822
Age	−0.040 *	0.01	−0.039 *	0.012
Marital Status (Unmarried, Living Apart, Divorced, Widowed = 0)				
Married but Living Apart	0.121	0.886	0.127	0.884
Married and Cohabiting	0.035	0.886	0.016	0.949
Informal Social Support			−0.499 *	0.036
N	509		509	
R^2^	0.036		0.043	

Note: Model 1 includes only control variables, whereas Model 2 additionally includes informal social support. * *p* < 0.05, ** *p* < 0.01.

**Table 5:** Mediation Path and Direct Effect Results for Life Satisfaction.

Explanatory Variables	Model 1		Model 2	
	β	*p*	β	*p*
Gender (Male = 0)				
Female	0.427 *	0.014	0.380 *	0.032
Household Registration Status (Agricultural = 0)				
Non-agricultural	0.068	0.648	0.085	0.704
Educational Attainment	−0.176	0.432	−0.159	0.484
Place of Residence (Rural = 0)				
Urban	−0.273	0.215	−0.276	0.211
Age	0.038 **	0.004	0.038 **	0.004
Marital Status (Unmarried, Living Apart, Divorced, Widowed = 0)				
Married but Living Apart	0.527	0.510	0.571	0.491
Married and Cohabiting	−0.055	0.798	−0.028	0.896
Depression Risk	−1.211 ***	0.000	−1.183 ***	0.000
Informal Social Support			0.385	0.059
N	509		509	
R^2^	0.045		0.048	

Note: Model 1 includes depression risk only in addition to the control variables, whereas Model 2 further includes informal social support on the basis of Model 1. * *p* < 0.05, ** *p* < 0.01, *** *p* < 0.001.

**Table 6:** KHB Decomposition Results for the Mediation Effect.

Effect	Estimate	SE	95% CI Lower	95% CI Upper	Relative Effect (%)
Indirect Effect	0.1165	0.0572	0.0043	0.2286	23.25%
Direct Effect	0.3845	0.2036	−0.0144	0.7835	76.75%
Total Effect	0.5010	0.2031	0.1029	0.8991	100.00%

Note: The KHB method was used to decompose the total effect into direct and indirect effects. SE = standard error; CI = confidence interval.

## Data Availability

The data used in this study primarily come from the China Health and Retirement Longitudinal Study (CHARLS). Open source: https://charls.charlsdata.com/pages/data/111/en.html. (accessed on 30 March 2026).
